# Identifying discriminative features for diagnosis of Kashin-Beck disease among adolescents

**DOI:** 10.1186/s12891-021-04514-z

**Published:** 2021-09-18

**Authors:** Yanan Zhang, Xiaoli Wei, Chunxia Cao, Fangfang Yu, Wenrong Li, Guanghui Zhao, Haiyan Wei, Feng’e Zhang, Peilin Meng, Shiquan Sun, Mikko Juhani Lammi, Xiong Guo

**Affiliations:** 1 School of Public Health, Xi’an Jiaotong University, Key Laboratory of Trace Elements and Endemic Diseases, National Health Commission of the People’s Republic of China, Xi’an, Shaanxi P.R. China; 2 School of Mathematics and Statistics, Xi’an Jiaotong University, Xi’an, Shaanxi P.R. China; 3 Institute of Disaster Medicine, Tianjin University, Tianjin, P.R. China; 4 Department of Health Statistics, College of Public Health, Zhengzhou University, Zhengzhou, P. R. China; 5 Department of Medical Imaging, The First Affiliated Hospital of Xi’an Jiaotong University, Xi’an, Shaanxi P. R. China; 6 Xi’an Honghui Hospital, Health Science Center of Xi’an Jiaotong University, Xi’an, Shaanxi P.R. China; 7 Department of Integrative Medical Biology, University of Umeå, 90187 Umeå, Sweden

**Keywords:** Kashin-Beck disease, Machine learning algorithms, Feature selection, Adolescents, Diagnosis

## Abstract

**Introduction:**

Diagnosing Kashin-Beck disease (KBD) involves damages to multiple joints and carries variable clinical symptoms, posing great challenge to the diagnosis of KBD for clinical practitioners. However, it is still unclear which clinical features of KBD are more informative for the diagnosis of Kashin-Beck disease among adolescent.

**Methods:**

We first manually extracted 26 possible features including clinical manifestations, and pathological changes of X-ray images from 400 KBD and 400 non-KBD adolescents. With such features, we performed four classification methods, i.e., random forest algorithms (RFA), artificial neural networks (ANNs), support vector machines (SVMs) and linear regression (LR) with four feature selection methods, i.e., RFA, minimum redundancy maximum relevance (mRMR), support vector machine recursive feature elimination (SVM—RFE) and Relief. The performance of diagnosis of KBD with respect to different classification models were evaluated by sensitivity, specificity, accuracy, and the area under the receiver operating characteristic (ROC) curve (AUC).

**Results:**

Our results demonstrated that the 10 out of 26 discriminative features were displayed more powerful performance, regardless of the chosen of classification models and feature selection methods. These ten discriminative features were distal end of phalanges alterations, metaphysis alterations and carpals alterations and clinical manifestations of ankle joint movement limitation, enlarged finger joints, flexion of the distal part of fingers, elbow joint movement limitation, squatting limitation, deformed finger joints, wrist joint movement limitation.

**Conclusions:**

The selected ten discriminative features could provide a fast, effective diagnostic standard for KBD adolescents.

**Supplementary Information:**

The online version contains supplementary material available at 10.1186/s12891-021-04514-z.

## Introduction

Kashin-Beck disease (KBD), a harmful endemic disease, affects more than 567.6 thousand patients and according to the “China Health and Family Planning Statistical Yearbook 2016”, could potentially threaten more than 1.16 million individuals in 377 counties from 13 provinces in China [1]. In addition, KBD cases have also been reported in the Eastern Siberia of Russia, and North Korea [2]. It is typically characterized by enlarged, deformed and shortened joints in the extremities, causing severe disabilities and disease burden [3, 4].

Diagnosing adolescents KBD is still a challenging task, and the omission diagnostic rate of adolescents KBD is more than 11.2% [5]. Currently, the national diagnostic criteria for KBD (WS/T207-2010) is revised on the basis of previous diagnostic criteria for Kashin-Beck Disease (GB16003-1995). The previous diagnostic criteria (GB16003-1995) focused on both clinical symptoms and X-ray alterations of hands. However, the current diagnostic criteria for KBD (WS/T207-2010) which emphasizes the importance of pathological changes of finger joints is a simpler and more convenient criteria for epidemiological surveillance and fast diagnosis [6]. Considering that KBD affects multiple joints in the whole body and the clinical manifestations of KBD among adolescents varies in individuals. Thus, single clinical manifestations could not provide sufficient evidence for KBD diagnosing. The available evidences indicate that even though single clinical manifestations, and X-ray pathological changes are strongly correlated with KBD diagnosis, they do not show effective, strong diagnostic performance on their own [7]. Therefore, it is crucial to find a cluster of features with high specificity and sensitivity for KBD among adolescents. In addition, the doctor’s experience is crucial in KBD diagnosis. However, most patients live in rural villages where doctors in the county-level hospitals lacked the necessary diagnosis experience. Some patients need to be transported to the cities for more specific consultation, which increase the overall cost of consultation. Therefore, a standard diagnostic method, contains a group of highly specific features with high sensitivity and specificity is warranted in order to KBD diagnosis among adolescents.

Machine learning algorithms (MLAs) have been widely applied in disease diagnosis and outcome predictions in recent years [8–10]. Compared with traditional data mining methods, the key advantage of using MLAs is its ability to process large amount of data in short time, uncovered new information and profiles of underlying relationships between databases [11]. Random forest algorithm (RFA), artificial neural network (ANN), support vector machines (SVMs) and linear regression (LR) are common algorithms of MLs. RFA is a substantial modification of bagging algorithms with the ability to process several possibly predictive variables which are interrelated in complex ways by reducing bias, avoiding overfitting and tolerating outliers [12, 13]. ANNs are modelled after the structure and behavior of human brain where each individual input variable is a “neuron”. An output outcome will obtain from measuring and processing the input variables after numerous rounds of learning events [14, 15]. Comparing to traditional linear regression(LR) ANN models are good at capturing nonlinear relationships between dependent and independent variables [16]. Support vector machines (SVMs) are a set of related supervised learning methods for classification, regression and ranking [17]. Therefore, one of the aims of this study is to compare the diagnosis efficacies of these methods.

Feature selection could offer support for machine learning tasks and it is applied to identify important feature variables from a large number of feature variables. Through feature selection, irrelative, redundant and noise data could be filtered and the accuracy of the classification could be improved [18, 19]. According to the relationship with the learning method, there are three categories of feature selection method, including filters, embedded methods and wrappers [20]. Considering there are many feature selection algorithms, we chose four representative methods including RFA, Max-relevance and Min-Redundancy (mRMR), support vector machine recursive feature elimination (SVM-RFE) and relief for each category for identifying discriminative features for KBD diagnosis.

To our knowledge, machine learning methods have not been reported in KBD diagnosis. In this study, 26 features including clinical manifestations, and pathological changes of X-ray images from 800 adolescents (400 confirmed KBD subjects and 400 non-KBD subjects) were extracted. Different machine learning algorithms including RFA, ANNs, SVM and LR were applied to build calssification models and the predictive efficacy of them were compared. More importantly, four feature selection algorithms were applied and we selected 10 discriminative features from 26 features. These 10 features with high sensitivity and specificity could provide a fast, effective diagnostic method for KBD diagnosis among adolescents.

## Methods

### Study population and sample size

The study was approved by Ethics Committee of Xi’an Jiaotong University, Xi’an, Shaanxi, China. All adolescents were at age between 5 to 16 years old and were from Linyou County and Bin County, two severely-affected endemic areas for KBD in Shaanxi province. Adolescents with any cartilage abnormalities, such as osteoarthritis (OA), rheumatoid arthritis (RA), rickets, or achondroplasia were excluded from the study sample. A balanced data set (1:1) including 400 KBD and 400 non-KBD adolescents were included in the study.

### Data collection

**Fig. 1:**
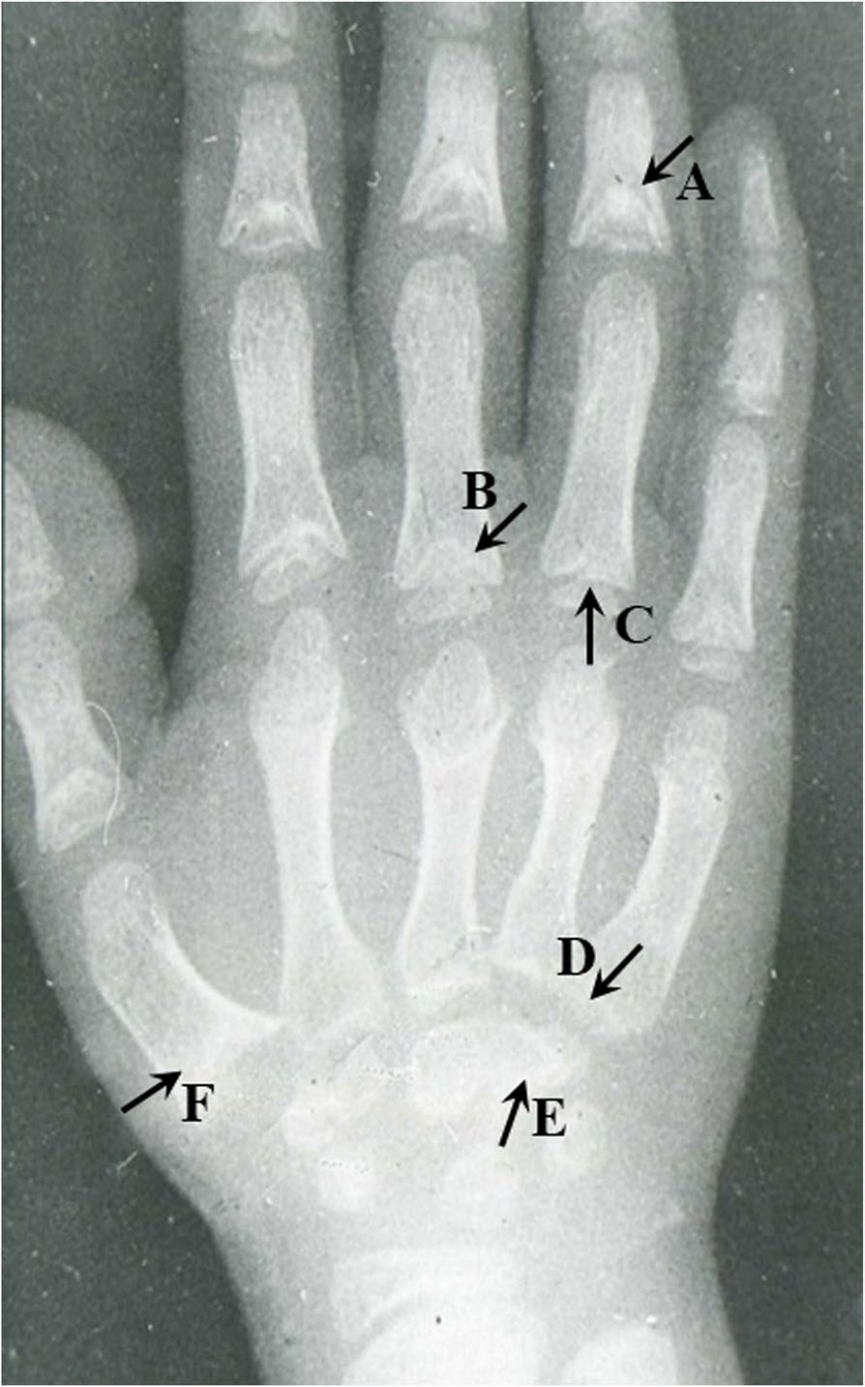
Examples of X-ray pathological changes of an eight-year old KBD boy. **A** A large defect with cone shaped showed in metaphysis alterations. There is an early closure of epiphysis line; **B** A large defect in metaphysis; **C** Cone shaped epiphysis; **D** Sclerosis in bottom of metacarpal bone; **E** Irregular marginal with sclerosis in carpal; **F** A large defect with sclerosis in carpal

Anteroposterior radiographs of the right hand of each subject were taken to observe the pathological alterations of metaphysis, distal end of phalanges, epiphysis, and carpals. Well-trained and experienced radiologists took the radiographs and followed the standard operating procedures strictly. X-ray pathological changes were extracted by two experienced orthopedic surgeons. The diagnostic criteria of X-ray radiographs for KBD are shown in Supplementary data 1, and an example of pathological X-ray radiograph changes of KBD is shown in Fig. 1. In addition, clinical symptoms were also checked by orthopedic surgeons. The examination checklist and evaluation standard were shown in Supplementary data 2. Finally, 26 features were extracted according to the evaluation standard (as lay-out in Table 1). KBD was diagnosed by three experienced experts according to X-ray pathological changes, clinical manifestations following the national diagnostic criteria (WS/T207-2010).

**Table 1:** List of extracted 26 features included in this study

**Clinical manifestations**	**X-ray pathological changes**
Joint pain	Metaphysis
Morning stiffness	Distal end of phalanges
Joint friction sound	Epiphysis
Dwarfism	Carpals
Short humerus	
Short fingers	
Flexion of the distal part of fingers	
Wrist joint movement limitation	
Elbow joint movement limitation	
Shoulder joint movement limitation	
Ankle joint movement limitation	
Knee joint movement limitation	
Squatting limitation	
Enlarged finger joint	
Enlarged elbow joint	
Enlarged knee joint	
Enlarged ankle joint	
Deformed finger joint	
Deformed wrist joint	
Deformed elbow joint	
Deformed knee joint	
Deformed ankle joint	

### Classification methods overview

We performed four existing classification methods, i.e., random forest algorithm (RFA), artificial neural network (ANNs), support vector machine (SVM) and logistic regression (LR), to predict the disease status (i.e., KBD or normal). The classification models were trained with default parameter settings companying with four feature selection methods (i.e., RFA, mRMR [21], SVM-RFE [22] and Relief [23]), which are falling into three categories, i.e., wrappers, embedded methods, and filters [24–26]. All methods were implemented by Python (Version 3.6.10) within sklearn framework (v 0.23.1). The performance of all four classification methods were evaluated by fivefold cross validation (5-CV) over four popular measures, i.e., sensitivity, specificity, accuracy, and the area under the ROC curve (AUC).

### Random forest algorithm (RFA)

The first classification method we performed in our analysis is random forest algorithm (RFA) [27], which is an ensemble learning method for KBD diagnosis purpose by constructing a multitude of decision trees (Supplementary Figure 1). We performed the RFA with *ensemble. RandomForestClassifier* function to construct RFA object; then utilized *fit* function to train the model, in which the training data sets and its corresponding class labels (i.e., disease status, KBD or normal) as inputs; finally carried out the *predict* function to predict the disease status with testing data sets. We performed various experiments to determine the optimal parameter, the number of variables randomly sampled as candidates at each split *m*_*try*_, and the number of trees ntree (Supplementary Figure 2), and finally, *m*_*try*_ = 3, and ntree = 300 were used in the following analyses.

### Artificial neural networks (ANNs)

There were three layers in ANNs classification models, an input layer, a hidden layer and an output layer [28]. The scheme of classification models using ANN was showed in Supplementary Figure 3 (F. S3.). For convenience, *neural_network.MLPClassifier* was implemented as ANNs classification model. By *GridSearchCV* which provide convenience for finding optimal parameter, there was only one hidden layer and the number of neurons was 5. In addition, the activation function was set as *Relu*. Learning rate was set as adaptive. *Lbfgs* was taken as optimization algorithm.

### Support vector machine (SVM)

Non-linear SVM algorithm was applied in this study. The hyperplane in non-linear SVM algorithm used kernel function to transform the decision function in the low dimensional plane. We establish non-linear SVM model by using *svm.SVC*, then other training and prediction steps is the same as RFA. For chosen of the kernel function and the coefficient (gamma) of it, with the help of *GridSearchCV* which provide convenience for finding optimal parameter, different settings are tried. Finally, we adopt *rbf* kernel and gamma value is set as the inverse of the number of features included. The fivefold cross-validation are also implemented for the propose of stabilized results.

### Logistic regression (LR)

Logistic regression algorithm adds a Sigmoid (for binary classification) or Softmax (for multi-classification) based on linear regression to solve dichotomous classification task. In this study, *Linearmodel.LogisticRegression* was applied and fit function was also implemented for training process*.* Predict labels and probabilities are available. Similar to SVM, *GridSearchCV* determines optimal parameter, and optimization algorithm was *lbfgs* and none penalties were chosen.

## Results

### General characteristic of study samples

Eight hundred adolescents (400 KBD and 400 non-KBD) were recruited. General characteristics of all study subjects were demonstrated in Table 2. There were significant differences of the age distribution between KBD and non-KBD group (${\chi }^{2}$ = 343.17, *p* < 0.001). Gender of the two groups showed no statistical differences (${\chi }^{2}$ = 0.18, *p* = 0.669). For clinical manifestations, significant differences between KBD and non-KBD group were observed in clinical grading of KBD, joint pain, short fingers, flexion of the distal part of fingers, wrist joint movement limitation, elbow joint movement limitation, ankle joint movement limitation, knee joint movement limitation, squatting limitation, enlarged finger joint, enlarged elbow joint, enlarged ankle joint, and deformed joints. In addition, more adolescents in KBD group showed pathological X-ray images in metaphysis alterations and distal end of phalanges alterations than those in non-KBD group and the differences were statistically significant.

**Table 2:** Characteristics of study subjects

**Variables**	**KBD (*n* = 400)** **n (%);**	**Non-KBD (*n* = 400)** **n (%);**	**χ2(*t*)**	***P* value**
**Age**				
< 6	5 (1.25)	23 (5.75)	343.17	< 0.001^***^
6 ~ 10	84 (21.00)	326 (81.50)		
10 ~ 14	258 (64.50)	48 (12.00)		
> 14	53 (13.25)	3 (0.75)		
**Male**	228 (57.00)	222 (55.00)	0.18	0.669
**Clinical grading**			-	-
I°	326 (81.50)	-		
II°	70 (17.50)	-		
III°	4 (1.00)	-		
**Clinical manifestations**				
Joint pain	13 (4.18)	4 (1.30)	4.58	0.032^*^
Morning stiffness	4 (1.29)	-	2.16	0.142^a^
Joint friction sound	1 (0.30)	-	-	1.000^b^
Dwarfism	-	-	-	-
Short humerus	4 (1.29)	-	2.16	0.142^a^
Short fingers	9 (2.89)	-	6.93	0.008^**^
Flexion of the distal part of fingers	167 (41.75)	25 (6.25)	138.19	< 0.001^**^
Wrist joint movement limitation	14 (3.50)	-	14.25	< 0.001^**^
Elbow joint movement limitation	38 (9.50)	-	39.90	< 0.001^**^
Shoulder joint movement limitation	1 (0.25)	-	-	1.000^b^
Ankle joint movement limitation	49 (12.25)	2 (0.50)	46.26	< 0.001^**^
Knee joint movement limitation	6 (1.50)	-	8.36	0.040^*a^
Squatting limitation	92 (29.58)	4 (1.33)	92.01	< 0.001^**^
Enlarged finger joint	71 (17.75)	2 (0.50)	71.77	< 0.001^**^
Enlarged elbow joint	4 (1.00)	-	5.57	0.018^*a^
Enlarged knee joint	1 (0.25)	-	-	1.000^b^
Enlarged ankle joint	6 (1.5)	-	8.36	0.040^*a^
Deformed finger joint	20 (5.00)	2 (0.50)	15.14	< 0.001^**^
Deformed wrist joint	2 (0.50)	-	2.78	0.096^a^
Deformed elbow joint	5 (1.25)	-	6.96	0.008^*a^
Deformed knee joint	5 (1.25)	-	6.96	0.008^*a^
Deformed ankle joint	5 (1.25)	-	6.96	0.008^*a^
**X-ray pathological changes**				
Metaphysis	75 (24.11)	21 (7.00)	33.78	< 0.001^**^
Distal end of phalanges	190 (61.09)	53 (17.67)	120.22	< 0.001^**^
Epiphysis	4 (1.29)	-	2.16	0.124^a^
Carpals	10 (3.22)	3 (1.00)	3.60	0.058

^*^p < 0.05; ^**^p < 0.01

^a^2 cells (50.0%) have expected count less than 5. The minimum expected count is between 1 and 5. Likehood ratio is adopted

^b^2 cells (50.0%) have expected count less than 5. The minimum expected count is 0.49. Fisher’s Exact Test is adopted

### Prediction performance of classification models

Classification models applied four different algorithms i.e., RFA, ANNs, SVM and LR were built based on 26 features. The prediction performance of four models were listed in Table 3. All four models showed good predictive efficacy with accuracy ranged from 93.63 to 99.76% and AUC value ranged from 0.94 to 1.00. Among four models, classification models of RFA and ANNs showed better predictive efficacy with higher AUC value (1.00, 1.00) and accuracy (99.76%, 99.63%) than models based on based on LR (0.97, 96.50%) and SVM (0.94, 93.63%). RFA model presented highest sensitivity with 100.00% and model SVM had lowest with 88.64%. Sensitivities of LR model and ANNs were 96.20 and 99.86%, respectively. The specificity of four models including RFA, ANNs, SVM, and LR model were 99.22, 99.66, 98.51 and 96.89%, respectively. To conclude, RFA and ANNs models had the best comprehensive predictive efficacy with highest AUC values.

**Table 3:** Prediction efficacy of KBD among adolescents by different machine learning methods

Diagnostic Model	Sensitivity (%)	Specificity (%)	Accuracy (%)	AUC
RFA	100.00	99.22	99.63	1.00
ANNs	99.86	99.66	99.76	1.00
SVM	88.64	98.51	93.63	0.94
LR	96.20	96.89	96.50	0.97

Sensitivity = Predictive Positive/True Positive × 100%; Specificity = Predictive Negative/True Negative × 100%; Accuracy = (Predictive Positive + Predictive Negative)/(True Positive + True Negative) × 100%; AUC = Area under the receiver operating characteristic curve (ROC)

*RFA* Random forest algorithm, *ANNs* Artificial neural networks, *SVM* Support vector machine, *LR* Logistic regression

### Feature selection

In this study, four algorithms including RFA, mRMR, SVM-RFE and relief were applied to select discriminative features for KBD diagnosis. The importance ranking of 26 features ranked by different algorithms were presented in Table 4. The order from 1 to 26 represented the range from the most important to the least important. The rankings of 26 features in RFA and mRMR algorithm were the same. The top 10 features in four algorithms were the same even the order of them were slightly varied. These top 10 features were distal end of phalanges alterations, metaphysis alterations, elbow joint movement limitation, ankle joint movement limitation, flexion of the distal part of fingers, enlarged finger joints, squatting limitation, carpals alterations, wrist joint movement limitation and deformed finger joints.

**Table 4:** Comparison of ranking of the 26 features using different feature selection algorithms

**Ranking**	**RFA**	**mRMR**	**SVM-RFE**	**Relief**
1	Distal end of phalanges alterations	Distal end of phalanges alterations	Elbow joint movement limitation	Squatting limitation
2	Metaphysis alterations	Metaphysis alterations	Distal end of phalanges alterations	Flexion of the distal part of fingers
3	Elbow joint movement limitation	Elbow joint movement limitation	Flexion of the distal part of fingers	Metaphysis alterations
4	Ankle joint movement limitation	Ankle joint movement limitation	Squatting limitation	Distal end of phalanges alterations
5	Flexion of the distal part of fingers	Flexion of the distal part of fingers	Metaphysis alterations	Elbow joint movement limitation
6	Enlarged finger joints	Enlarged finger joint	Ankle joint movement limitation	Ankle joint movement limitation
7	Squatting limitation	Squatting limitation	Enlarged finger joint	Enlarged finger joint
8	Carpals alterations	Carpals alterations	Knee joint movement limitation	Carpals alterations
9	Wrist joint movement limitation	Wrist joint movement limitation	Deformed finger joint	Wrist joint movement limitation
10	Deformed finger joints	Deformed finger joint	Carpals alterations	Deformed finger joint
11	Knee joints movement limitation	Knee joint movement limitation	Enlarged elbow joint	Knee joint movement limitation
12	Joint pain	Joint pain	Wrist joint movement limitation	Joint pain
13	Enlarged Elbow joints	Enlarged elbow joint	Dwarfism	Enlarged elbow joint
14	Short humerus	Short humerus	Deformed elbow joint	Short humerus
15	Enlarged ankle joint	Enlarged ankle joint	Joint pain	Enlarged ankle joint
16	Deformed knee joint	Deformed knee joint	Deformed ankle joint	Deformed knee joint
17	Deformed ankle joint	Deformed ankle joint	Deformed knee joint	Deformed ankle joint
18	Deformed elbow joint	Deformed elbow joint	Enlarged ankle joint	Deformed elbow joint
19	Epiphysis alterations	Epiphysis alterations	Epiphysis alterations	Epiphysis alterations
20	Dwarfism	Dwarfism	Short humerus	Dwarfism
21	Joint friction sound	Joint friction sound	Shoulder joint movement limitation	Joint friction sound
22	Short fingers	Short fingers	Morning stiffness	Short fingers
23	Shoulder joint movement limitation	Shoulder joint movement limitation	Deformed wrist joint	Shoulder joint movement limitation
24	Enlarged knee joint	Enlarged knee joint	Deformed knee joint	Deformed knee joint
25	Deformed wrist joint	Deformed wrist joint	Short fingers	Deformed wrist joint
26	Morning stiffness	Morning stiffness	Joint friction sound	Morning stiffness

**Fig. 2:**
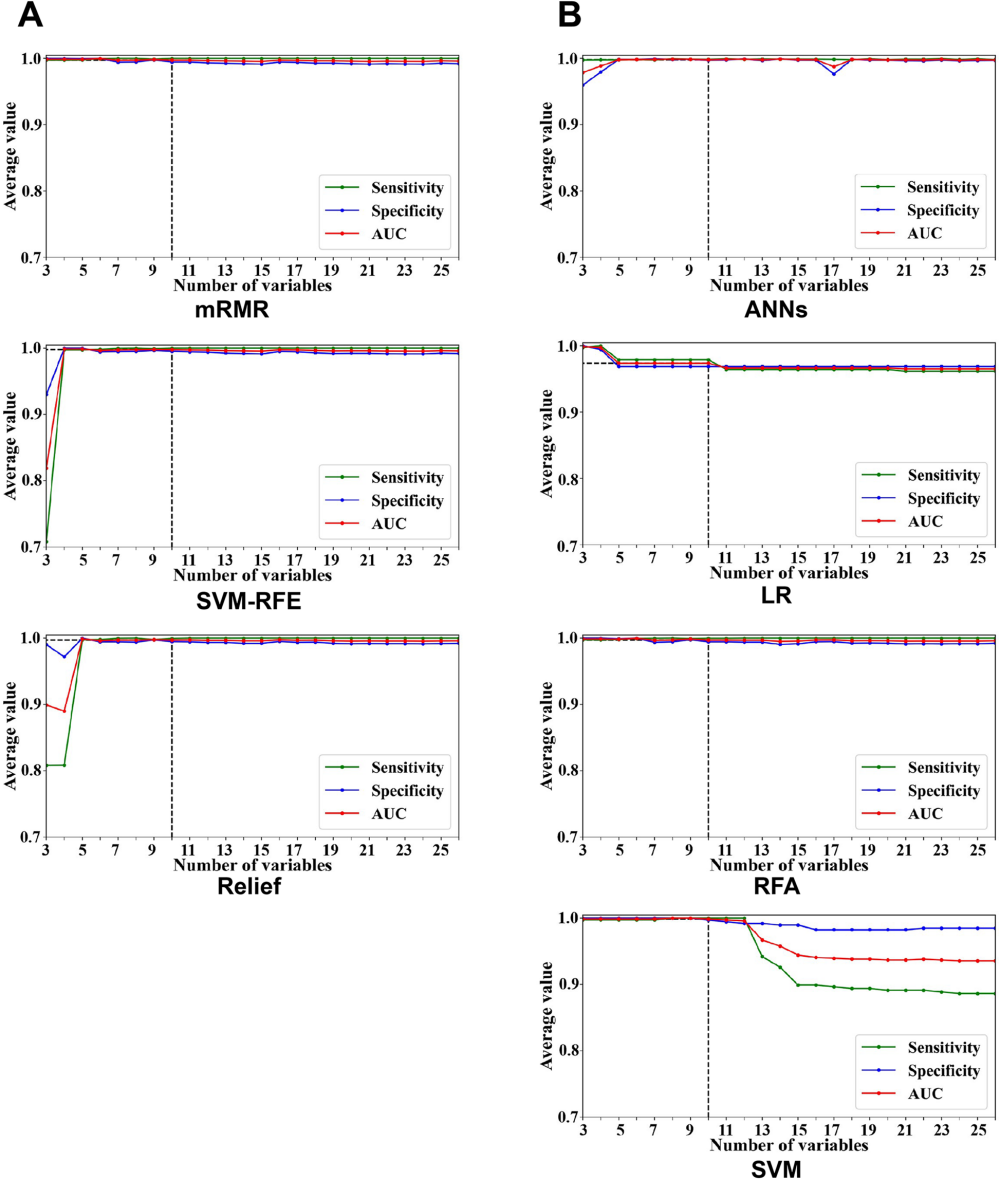
Diagnostic efficacies with respect to the number of features. **A** Prediction efficacies in RF predictive models in accord to the importance ranking of 26 features measured by mRMR, SVM-RFE and relief. **B** Prediction efficacies in RF, ANNs, SVM and LR predictive models following the importance ranking of mRMR algorithm

To assess the predictive performance of selected features, prediction performance with respect to the number of selected features were showed in Fig. 2. We firstly applied RFA which showed the best prediction efficacy among four classification models to test the prediction performance with the different number of selected features (Fig. 2A). We found that the predictive efficacy was stable and was the best when the number of features was ten. Then we tested the predictive efficacy using different ML classification models according to the ranking of mRMR (Fig. 2B). Even the trends of four ML models were slightly different, high AUC values were showed when the number of selected features were ten. In model of LR and SVM, the predictive efficacy peaked at where the number of features were up to 10. In conclusion, the top 10 selected features out of 26 features showed high predictive efficacy regardless of the chosen of classification models and feature selection methods.

## Discussion

To the best of our knowledge, this is the first time that the MLs and feature selection algorithms have been used to aid diagnose KBD among adolescents. In this study, we applied feature selection algorithms and found 10 out of 26 features with high sensitivity and specificity for KBD diagnosis among adolescents.

In this study, four algorithms which represente three categories of feature selection methods were applied to select the discriminative features for KBD diagnosis among adolescents (Table 4). We found that pathological changes of X-ray images including distal end of phalanges alterations, metaphysis alterations, and carpals alterations, clinical manifestations including ankle movement limitation, enlarged finger joints, flexion of the distal part of fingers, elbow movement limitation, squatting limitation, deformed finger joints, wrist movement limitation were the top 10 diagnostic features of KBD regardless of the feature selection methods. In order to confirm this finding, predictive efficacies respect to the number of features were also calculated in different classification models (Fig. 2). We found that the predictive performace of different models were stable and with high sensitivity and specificity when the number of features were 9 and 10. These results indicated that these 10 features could be discriminative features for KBD diagnosis among adolescents.

The previous diagnostic criteria (GB16003-1995), emphasized the importance of X-ray alterations in distal end of phalanges, metaphysis, epiphysis and carpals (Supplementary data 1) for KBD diagnosis [29–31]. In our study, all four feature selection algorithms revealed that alterations of distal end of phalanges, metaphysis, carpals were discriminative features of X-ray images for diagnosis of KBD among adolescents. A previous study highlighted that the abnormities of carpal bones was helpful for KBD diagnosis among children and there was also a correlation between the abnormities of carpals and the severity of KBD [31]. In this study, marginal interruption, irregularity with sclerosis, defect, impaired development, deformed and absence of carpals were defined as positive X-ray alterations (Supplementary data 1). Even though the distribution difference of carpals alterations among KBD and non-KBD adolescents were not statistically significant (Table 2; χ^2^ = 3.599, *P* > 0.05), features selection algorithms still highlighted the importance of carpals alterations in X-ray images in KBD diagnosis. However, the epiphysis alterations in KBD diagnosis were not addressed in our findings. Alterations of epiphysis was not a sensitive feature for KBD diagnosis among adolescents. The reason behind this was that the vascularity and metabolism were not as strong as that in metaphysis. Therefore, the epiphysis was less sensitive to damages than metaphysis. Usually, alterations of epiphysis were indicators of irreversible damages of cartilage [32]. In addition, the findings of this study also revealed that ankle movement limitation was a significant feature for KBD prediction among adolescents. In our study, nearly 12.25% of adolescents with KBD (49 of 400) presented ankle movement limitation, while only 0.5% (2 of 400) of healthy adolescents reported ankle movement limitation. Previous studies reported that nearly 68.8% KBD adult patients showed abnormal ankle radiographs, pathological changes of X-ray images including talus, calcaneus, navicular bone and distal tibia [33]. Until now, the diagnostic value of ankles had not been emphasized. This new finding suggests that the diagnostic value of ankles, including clinical manifestations and radiological changes, might be significant to KBD diagnosis. Even the prevalence of KBD among adolescents is much lower than that in adults, we believe that this cluster of features selected based on importance ranking also apply to diagnosis of KBD adults. KBD patients start showing symptoms during adolescents and symptoms aggravates with age. Most adult KBD patients share similar clinical symptoms with adolescent patients while the X-ray alterations were a little different between them since skeletal development. Among these ten features, only three out of ten of them were X-ray alterations. We believe these ten features also apply for adult KBD patients.

RFA, ANNs, SVM and LR were applied to to develop different classification models and predictive performance of them were compared to choose the most suitable classification model for KBD diagnosis among adolescents. Among four classification models, RFA showed the best predictive efficacy with highest AUC value (1.00). Studies have reported that RFA was an optimal choice for building predictive or diagnostic model with its high diagnostic efficacy [9, 34]. Some scholars believed that ANNs are inherently “opaque and lack interpretability”; its classification process akin to “black box” and its input variables cannot be adjusted independently at each intermediate step [35]. While in a random forest model, there are many decision trees, and each tree is built based on a randomly selected subset from the training data and a random subset of input variables. The variables can be ranked at each decision tree and a final decision will be made by voting these randomly generated subsets [13].

There are some limitations of this study. First, we only used “KBD” or “non-KBD” as output results in all three models, without considering the disease stages of KBD. In order to give more accurate diagnosis for adolescents KBD, more specific models focusing on stages of disease with larger training data should be developed. Second, we still spent some time reading X-ray images to extract 26 features before we started building classification model. Recent studies reported image recognition algorithms, such as conventional neural networks which could read radiographs to aid diagnosis [36–38]. In the future, a smarter diagnostic model which could read X-ray images combining with our diagnostic model is needed to provide fast, effective diagnostic method. Third, we excluded the subjects with OA and RA, whom present similar clinical manifestations and X-ray radiological changes with KBD. Considering that identifying these three kinds of diseases and classifying them is a daily work for orthopedists, it is very necessary to build a comprehensive model which could classify these three diseases based on symptoms and changes of radiological images. Even though, our study still gave us a hint that MLs would be helpful and be generalized by increasing sample size and accuracy of algorithms, multiple computer-based methods and algorithms can integrate to establish a more intelligent, specific model to provide a more accurate diagnosis.

## Conclusions

We calibrated classification models based on MLs in order to integrate clinical manifestations and radiograph alterations to aid diagnosis of KBD among adolescents. We found 10 out of 26 discriminative features with high sensitivity and specificity for KBD diagnosis among adolescents. These features could provide a quick, effective diagnostic methods for KBD.

## Supplementary Information

**Additional file 1.** The X-ray radiograph alterations among KBD adolescents.

**Additional file 2.** The examination list of clinical symptoms and diagnostic criteria.

**Additional file 3: Figure S1.** The scheme of random forest tree algorithm. There were 300 decision trees in RF classficaiton model. The final classification outcome of each sample was decided by voting on the most popular classification of these 300 trees.

**Additional file 4: Figure S2.** Out of bag (OBB) error rate to assess the quality of random forest algorithm to predict KBD. When m_*try*_ was set as 3, the OBB error rate was decrease quickly and become stable at where ntree was 300.

**Additional file 5: Figure S3.** The scheme of artificial neural networks. In this study, there were 26 input variables and hidden neurons were 5.

## Data Availability

The datasets used and/or analyzed during the current study are available from the corresponding author on reasonable request.

## Abbreviations

MLs: Machine learning algorithms
KBD: Kashin-Beck disease
RFA: Random forest algorithm
ANNs: Artificial neuron networks
SVM: Support vector machine
SVM-RFE: Support vector machine recursive feature elimination
mRMR: Max-Relevance and Min-Redundancy
ROC: Receiver operating characteristic curve
AUC: Areas under the receiver operating characteristic curve
OA: Osteoarthritis
RA: Rheumatoid arthritis
OBB: Out of Bag
