# Supplementary material for: Identifying discriminative features for diagnosis of Kashin-Beck disease among adolescents
Source: BMC Musculoskelet Disord. 2021 Sep 18;22:801. doi: 10.1186/s12891-021-04514-z (PMC8449456; doi:10.1186/s12891-021-04514-z)
Supplement: Supplementary file 1 — Additional file 1. The X-ray radiograph alterations among KBD adolescents. [file 12891_2021_4514_MOESM1_ESM.docx]

**Supplementary data 1**

**The X-ray radiograph alterations among KBD adolescents**

| **X-ray Image Alterations** | **Specific description** |
| --- | --- |
| **Metaphysis** | Blurred and interrupted; Bone trabecula disturbance |
|  | Thick marginal sclerosis; Defect |
| **Epiphysis** | Irregular marginal sclerosis; Blurred; Flattening; |
|  | Premature closure of epiphyseal line; Thinner |
|  | Cone shapes; Absence; Fragmented |
| **Distal end of phalanges** | Marginal spur; Irregular; Small defect with sclerosis; Bone trabecula disturbance |
|  | Marginal defect; Hyperostosis; Pouch-like changes; Calcification |
|  | Enlarged |
| **Carpals** | Marginal interruption; Irregularity with sclerosis |
|  | Defect; Impaired development; |
|  | Deformed; Absence; |

Note: Adolescents who are with one of the pathological changes is defined as “positive”.

The X-ray alterations were defined according to the published paper ‘Diagnostic, clinical and radiological characteristics of Kashin-Beck disease in Shaanxi Province, PR China’, published in *International Orthopaedics*, 2001.
