# Supplementary figures and images for: Identifying discriminative features for diagnosis of Kashin-Beck disease among adolescents
Source: BMC Musculoskelet Disord. 2021 Sep 18;22:801. doi: 10.1186/s12891-021-04514-z (PMC8449456; doi:10.1186/s12891-021-04514-z)

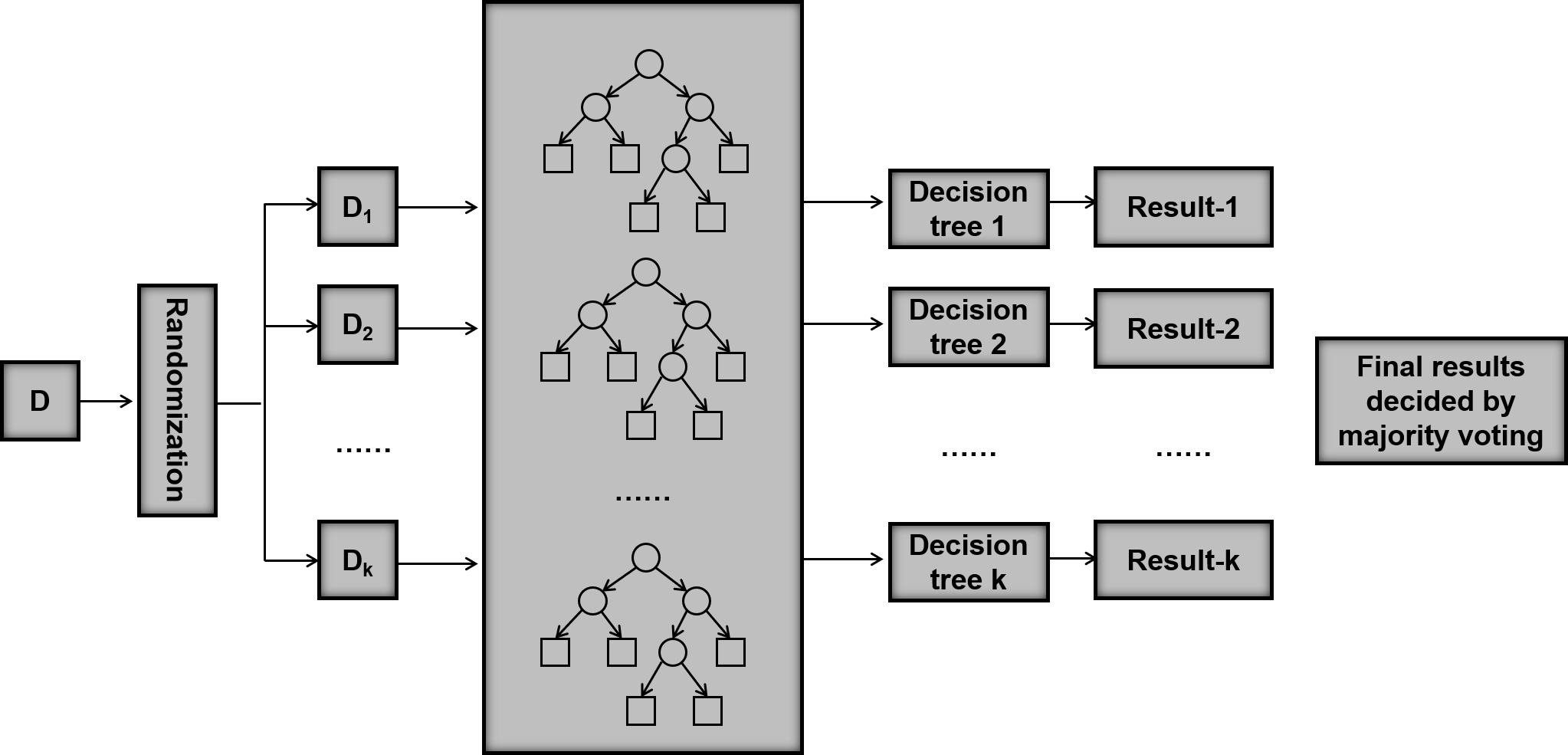

Supplement: Supplementary file 2 — Additional file 2. The examination list of clinical symptoms and diagnostic criteria. [file 12891_2021_4514_MOESM2_ESM.tif]

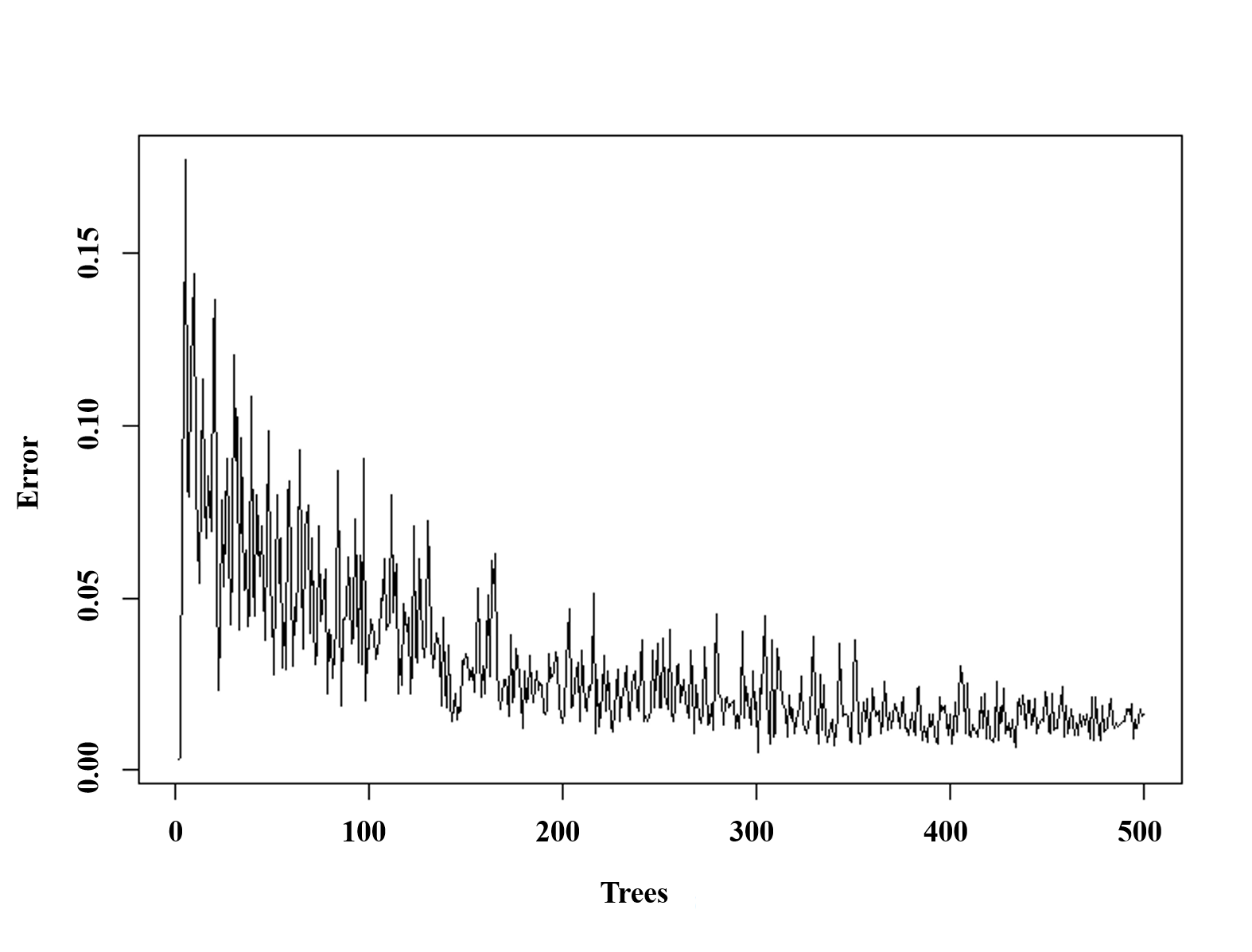

Supplement: Supplementary file 4 — Additional file 4: Figure S2. Out of bag (OBB) error rate to assess the quality of random forest algorithm to predict KBD. When mtry was set as 3, the OBB error rate was decrease quickly and become stable at where ntree was 300. [file 12891_2021_4514_MOESM4_ESM.tif]

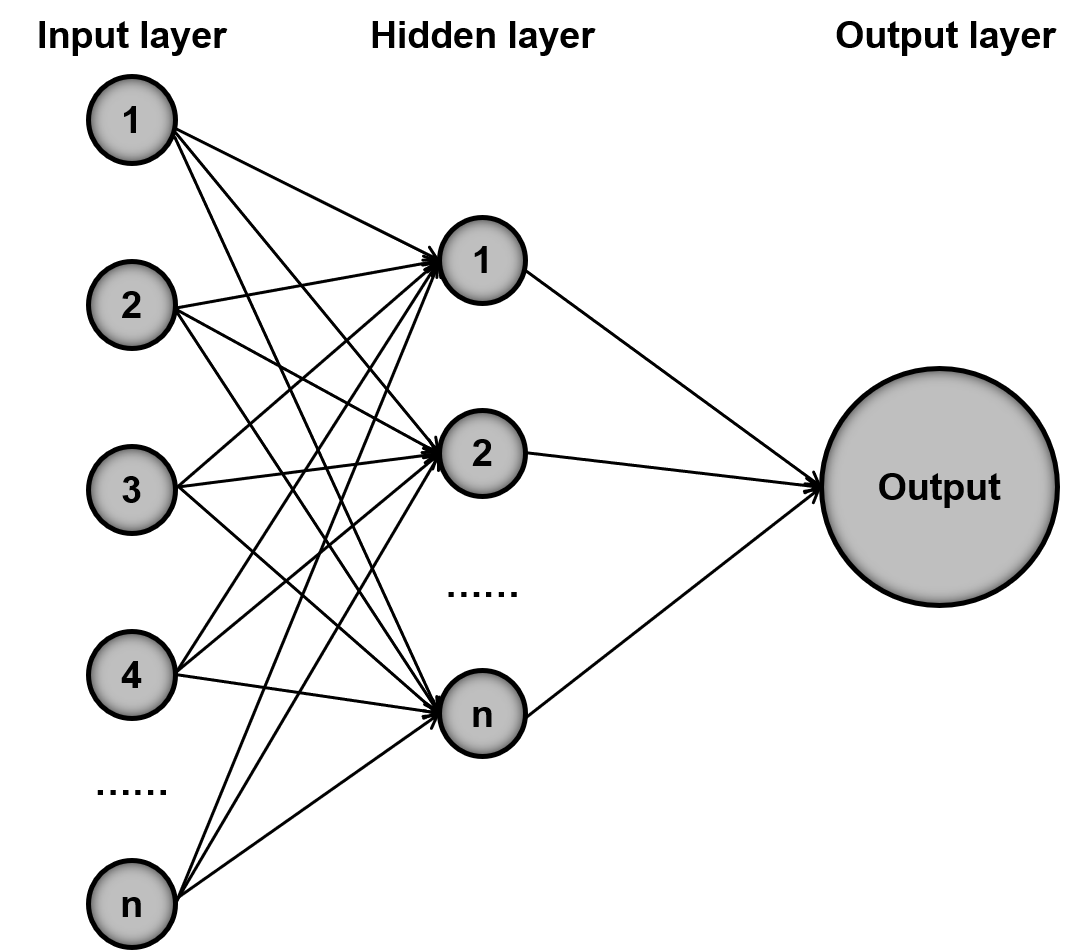

Supplement: Supplementary file 5 — Additional file 5: Figure S3. The scheme of artificial neural networks. In this study, there were 26 input variables and hidden neurons were 5. [file 12891_2021_4514_MOESM5_ESM.tif]
